# Growth and longevity in giant barrel sponges: Redwoods of the reef or Pines in the Indo-Pacific?

**DOI:** 10.1038/s41598-018-33294-1

**Published:** 2018-10-17

**Authors:** Emily C. McGrath, Lisa Woods, Jamaluddin Jompa, Abdul Haris, James J. Bell

**Affiliations:** 1 School of Biological Sciences, Victoria University of Wellington, P.O. Box 600, Wellington, 6140 New Zealand; 2 School of Mathematics and Statistics, Victoria University of Wellington, P.O. Box 600, Wellington, 6140 New Zealand; 3 Research and Development Centre on Marine, Coastal and Small Islands, Hasanuddin University, Makassar, Indonesia

**Keywords:** Conservation biology, Ecosystem ecology

## Abstract

Describing life history dynamics of functionally important species is critical for successful management. Barrel sponges (*Xestospongia* spp.) fill ecologically important roles on coral reefs due to their large size and water column interactions. Studies of Caribbean *X. muta* suggest they may be up to 1000 s of years old. However, nothing is known of barrel sponge growth rates outside the Caribbean. We assessed Indo-Pacific barrel sponge demography with a focus on specific growth rate (SGR), density, and mean volume across four sites of varying habitat quality. Four growth models were compared using Akaike’s Information Criterion using a multi-model inference approach. Age was extrapolated and validated based on sponge size on a shipwreck of known age. Sponges from different sites showed differences in density, volume gained, and mean volume, but not growth rates. Interestingly, SGRs were slightly slower than that of *X. muta*, yet growth models supported rapid growth; published estimates of comparably sized *X. muta* were over twice as old as Indo-Pacific sponges (53–55 as compared to 23 years of age, respectively), although extrapolation errors are likely to increase with sponge size. This suggests that barrel sponge growth rates in the Indo-Pacific might be more comparable to Pines rather than Redwoods.

## Introduction

Understanding the life history traits of an organism including growth, recruitment, and mortality are central to quantifying its contribution to ecosystem functioning^1^, as well managing species in response to environmental perturbations^2^. The size of an organism, and the population within which it resides, will likely affect the magnitude of its influence on other organisms^3^. Size is typically related to life-history processes such as mortality, growth and reproduction^4^, as well as its spatial competitiveness^5^ and ability to consume resources. However, these processes are not independent of the environment and are likely to be influenced by a range of abiotic and biotic factors^6^.

An organism’s lifespan, along with its population and individual growth rate, can potentially be used to predict its resilience to environmental disturbance or exploitation^7^. For example, long-lived organisms with small population sizes that have sporadic or infrequent recruitment, low fecundity and slow growth rates (k-strategies) are likely to be more sensitive to disturbance compared to fast growing, short-lived, highly fecund species with large population sizes (r-strategies). Therefore, accurate measures of growth, recruitment, mortality, and age-structure are needed to support appropriate conservation and management strategies^8^.

Sponges are one of the most ancient and simple metazoans that have evolved into an abundant, diverse, and ecologically important group in both marine and freshwater habitats^9,10^. Highly efficient particle retention, coupled with the ability to pump large quantities of water relative to their size, results in the potential to strongly modify water column characteristics by removing a large portion of available particulate food^11^ and dissolved organic carbon^12^. Sponges link water column productivity and the benthic community via benthic-pelagic coupling^13,14^, by facilitating carbon transport^15,16^, and nitrogen^15^ and silicon cycling^17^. Sponges therefore have a number of important functional roles on reefs and changes in sponge populations have the potential to impact ecosystem function^18,19^.

While sponges show a range of life-history strategies, some species are thought to be very long lived, with estimated lifespans ranging from decades to thousands of years old^20–24^. Among the largest known sponges, those in the genus *Xestospongia* can grow up to several meters in diameter^22^ and pump large quantities of water^25^. Until recently, Indo-Pacific barrel sponges were believed to solely include *X. testudinaria*, *X. exigua*, and *X. berguista*. However, recent genetic work in this region has revealed a potential cryptic species complex^26,27^; as such “*Xestospongia* spp.” will be used throughout. In some areas of the Indo-Pacific *Xestospongia* spp. are commonly found in dense population in sites of high sedimentation and low coral cover^28^. As such, *Xestospongia* spp. may be among largest remaining benthic invertebrates on reef systems where coral cover is declining. These sponges rarely stop pumping^25^ and are highly efficient in retaining picoplankton (62–97%), while also consuming dissolved organic carbon (DOC)^29^. Despite this highly efficient feeding, it has been suggested that Caribbean *X. muta* growth is variable and they could live to be hundreds or possibly even thousands of years old^22^. These features would be expected to render *X. muta* susceptible to environmental perturbations. However, it is currently unknown whether such characteristics are applicable to *Xestospongia* spp. in the Indo-Pacific.

Growth models are widely used to describe increases in size or volume over time, particularly in fisheries biology^30^. Choosing the appropriate models is critical as poor model selection may lead to errors in parameter estimation and subsequent inferences about growth dynamics^31^ and age/size estimations^1,32,33^. Rather than making an arbitrary choice *a priori* and identifying the “best” candidate model(s), multi-model inference (MMI) using model averaging can be used to estimate parameters from multiple or an entire set of candidate models in order to reduce selection uncertainty^32^. This method entails examining the fit of a range of candidate models to the data based on parsimony according to Akaike Information Criterion (AIC)^34,35^, allowing for robust comparisons between models which could not otherwise be compared^31^. MMI should be considered when Akaike weights (*w*_*i*_) support more than one model^32,36,37^. As sponges lack features comparable to otoliths and absolute size-at-age is difficult to quantify in slow growing species, MMI is expected to be particularly useful for reducing model selection uncertainty when estimating sponge growth.

While there has been considerable study of *Xestospongia muta* in the Caribbean, the demography of Indo-Pacific *Xestospongia* spp. has been poorly studied despite being widespread across the region and likely to fulfil similar functional roles. Understanding barrel sponge demography is particularly important given current trends of habitat degradation in the Indo-Pacific and elsewhere. Here the demography of *Xestospongia* spp. is examined by quantifying individual growth parameters and density across sites. A multi-model inference (MMI) approach with Akaike weights is used to model average four candidate growth models. Akaike differences were examined to select models of best fit and these models were used to estimate size-at-age, providing important insight into growth dynamics and potential resilience to environmental perturbations.

## Methods

### Study sites

**Figure 1:**
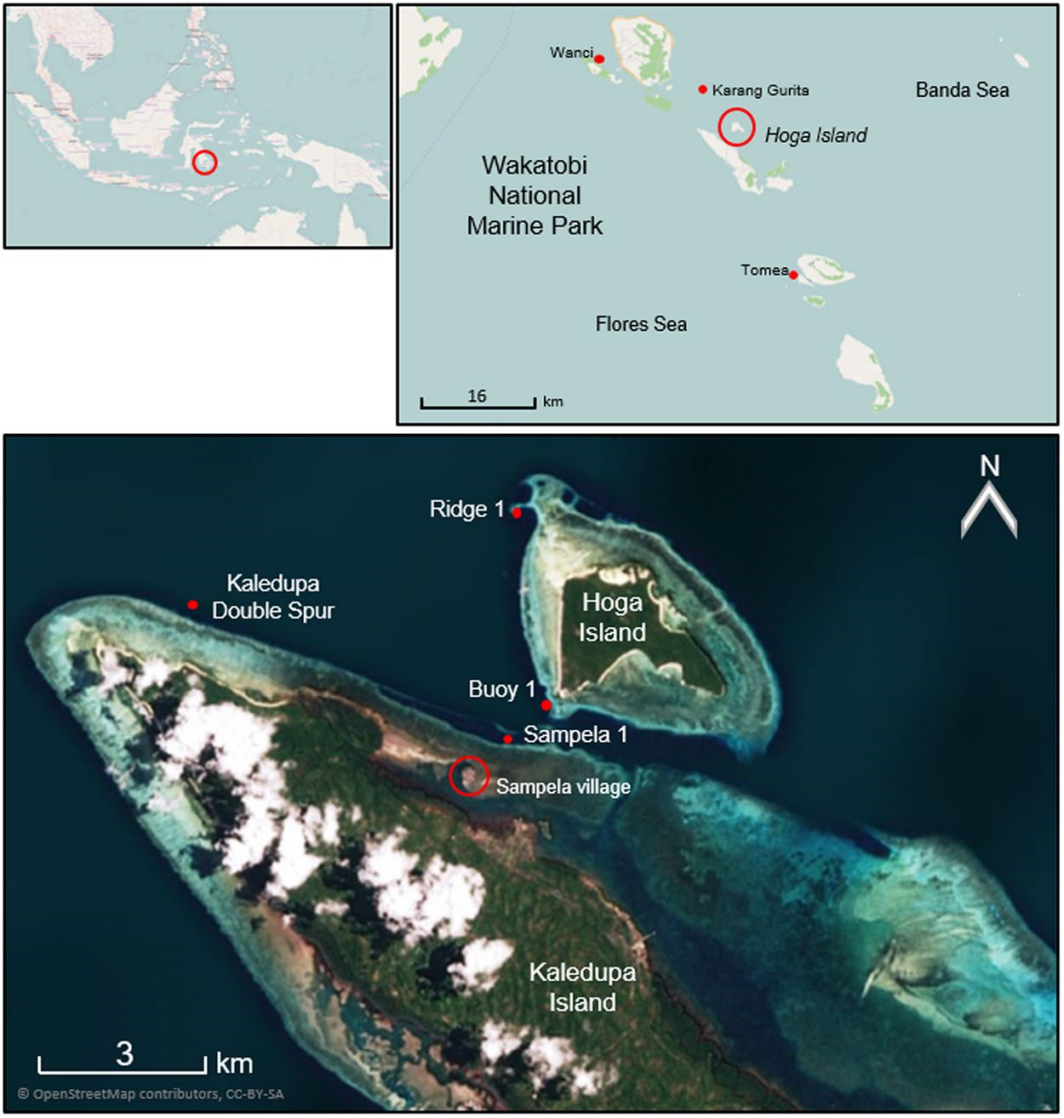
Map of the study area in relation to Indonesia and the Wakatobi Marine National Park, as well as the study sites (red dots) with the proximity to Kaledupa Island, Hoga Island and Sampela village (map backgrounds: © OpenStreetMap contributors, CC BY-SA).

Four ‘core’ sites were surveyed around Hoga Island in the Wakatobi Marine National Park (WMNP) between 2014 and 2016: Sampela 1, Ridge 1, Buoy 1, and Kaledupa Double Spur (Fig. 1). Data were collected from depths of 1 to 30 m. Abiotic and biotic characteristics of these sites are summarized from previous studies in Supplementary Table 1. Three further sites were surveyed in 2014 to estimate population densities in the wider WMNP: Karang Gurita, Wanci Harbor, and Tomea (Fig. 1). No environmental data were collected for the sites sampled in the wider WMNP, although previous studies have provided environmental descriptions^28^.

### Sponge volume measurement

Data were collected from June to August in 2014, 2015 and 2016. Sponge images were collected yearly from Sampela 1, Buoy 1, Ridge 1, and Kaledupa Double Spur, while wider WMNP sites (Wanci, Tomea, and Karang Gurita) were sampled in 2014. Each Hoga Island study site was mapped at the beginning of the study and all sponges were marked with a unique tag to facilitate subsequent re-identification. Digital images for stereo photogrammetric analysis were taken with a Fujifilm FinePix Real 3D W3 Digital Camera with corresponding underwater camera housing.

Volumetric measurements were calculated using stereo calibration and measurement software (CAL and PhotoMeasure) created by J. Seager (http://www.seagis.com.au); calibration procedures are described in the Supplementary Information. The use of stereo photogrammetry allows for accurate repeated 3D measurements in order to best calculate true external and spongocoel volume^38^, though the internal canal system remains difficult to quantify^22^. Furthermore, stereo photogrammetry allows for multiple measurements to be made for a variety of sponge parameters^38^. Volume was calculated by approximating geometric shapes for each sponge shape and corrected for spongocoel volume^22^ (described in the Supplementary Information). Due to the highly diverse morphologies of Indo-Pacific *Xestospongia* spp., sponges were categorized as either cylinder, barrel, sphere, inverted truncated elliptical cone, or frustrum of a cone. Spongocoels were categorized as either cylinders or inverted truncated elliptical cones (depending on the sponge) and volume was calculated accordingly. If sponge morphology changed over time the formulae were adapted as appropriate. The total number of sponges per site used for volumetric analyses were as follows: Buoy 1 (35), Kaldeupa Double Spur (14), Ridge 1 (16), and Sampela 1 (56).

### Barrel sponge demography

Specific growth rate (SGR) was calculated as the difference in sponge volume (cm^3^) divided by the two year interval between sampling events^22^ (2014–2016; Eq. 1)

1 $$SGR=({V}_{t}-{V}_{i})\,\ast \,{V}_{i}^{-1}/t$$

Negative SGRs were confirmed from photographs and were only removed from analyses were they severely damaged or demonstrating severe necrosis. Sponge density was calculated by dividing the total sponge number at each site by the total area sampled during timed roving diver swims of transects approximately 50 m wide (as quantified by pre-measured fin kicks): Buoy 1 (4,500 m^2^), Sampela 1 (5,450 m^2^), Kaledupa Double Spur (5,952 m^2^), Ridge 1 (5,238 m^2^), Wanci (3,500 m^2^), Tomea (11,880 m^2^), Karang Gurita (22,780 m^2^). Sponge density surveys were only conducted once for each site in 2014.

### Candidate models

Five growth models were used to investigate the growth of *Xestospongia* spp.: specialized von Bertalanffy, generalized von Bertalanffy, Gompertz, Richards, Tanaka (Supplementary Table 2). The Richards equation produced values identical to those in the generalized von Bertalanffy model and as such was removed to avoid model redundancy^36^.

### Data analysis

All statistical analyses were performed by SPSS v. 22 and in R (version 3.3.3) and plotted with SigmaPlot v. 11.0 and R. Data were tested for normality and homogeneity of variance; volume, density, and SGR were log_10_-transformed. Values are reported ± standard error (SE) throughout.

Sponges were grouped into depth ranges of 1–9.9, 10–19.9, and 20–30 m at each site^22^. A one-way analysis of covariance (ANCOVA) was used to examine the influence of depth on sponge growth, with initial volume as the covariate and depth (10, 20, 30 m) as a fixed factor. This relationship was not significant (one-way ANCOVA, F_2,99_ = 2.232, *P* = 0.089), and as such depth was removed from further analyses. One-way ANOVAs were used to examine the effect of site on specific growth rate, yearly gains in volume, and sponge density averaged across depths. A repeated measures two-way ANOVA was used to examine the effect of site and year on mean sponge volume. Specific growth rates (SGR) from 2014–2016 were used as it was the longest available time interval. Mean volume gain was only used for the 2015–2016 sampling event due to the small sample size of Ridge 1 sponges between 2014 and 2015. Significant effects were investigated further with Tukey post-hoc tests.

### Model selection

Size increment volume data ranging from 2014 and 2016 were fitted to the candidate growth models by nonlinear least-squares regression (nlsLM, R) using the Levenberg-Marquardt algorithm. Sponge volume data, corrected for spongocoel volume, was cube root transformed for model input, and the difference equation for each function was applied to the transformed data^22,33^; Initial analyses were separated by site (Buoy 1, n = 35; Kaledupa Double Spur, n = 14; Ridge 1, n = 16; Sampela 1, n = 56). Models were discarded from further analysis if they failed in predicting reliable asymptotic lengths, or those representative of realistic size estimates based on known sponge sizes^39^. After the growth rate at each site was considered independently by calculating unique Akaike weights, multi-model inference (MMI) with model averaging was utilized for each site (detailed in the Supplementary Information).Then, in order to quantify the influence of site on *Xestospongia* spp. growth rates, a second model analysis approach was taken. AIC_c_ values for each growth model were pooled across sites. The models with highest support were then averaged and compared across sites using an analysis of the residual sum of squares (ARSS). There was no statistically significant influence of site on growth (*P* = 0.4033) and as such the analyses were run again with all sites pooled for MMI and subsequent size-at-age extrapolation (n = 121).

### Size-at-age predictions

In order to retrospectively extrapolate *Xestospongia* spp. size-at-age when the actual age was unknown, the predicted size (volume) at time *t* was estimated for each growth function using parameter estimates from the size increment data. Integrated versions of the relevant growth functions were solved for *t*_0_ using *t* = 0 and the smallest sponge measured as size at *t*_0_^22^ (19.99 cm^3^). Given *t*_0_, size-at-age *t* is predicted for each growth model, and then weighted by *w*_*i*_ to obtain the model averaged estimate of size-at-age *t*. Values were then cubed to obtain size (volume [cm^3^])-at-age plots for all sites combined.

### Model validation

In order to validate our size-at-age estimates we used an opportunity where barrel sponges have settled on a shipwreck in northern Bali. The USAT Liberty was torpedoed in 1942 during WWII and ran onto shore (8°16′28.48″S; 115°35′35.02″E), where it rested until it sank in 1963 after the tremors associated with the eruption of Mount Agung caused the ship to slip into the sea. The wreck rests on a sand slope adjacent to a shallow reef (8–10 m) and runs from 5 m to 22 m at its base. In 2014 stereo photogrammetry (as described in the Methods above) was used to measure the volume of 10 haphazardly distributed sponges in an effort to provide an earliest possible date (1963) when *Xestospongia* spp. could have recruited to the wreck. We then used our size-at-age model to estimate the approximate age of the sponges on the wreck and compared these to the known maximum possible sponge age.

## Results

### Barrel sponge demography

*Xestospongia* spp. density varied significantly across sites around Hoga Island and across the wider Wakatobi Marine National Park (one-way ANOVA: F_2,7_ = 3.889, *P* = 0.008; n = 268). Sites with the highest sponge densities were Buoy 1, Sampela 1, and Karang Gurita, with densities of 0.017, 0.014, and 0.013 sponges m^−2^, respectively, followed by Ridge 1 (0.006 sponges m^−2^), Kaledupa Double Spur (0.005 sponges m^−2^), Wanci (0.004 sponges m^−2^), and Tomea (0.002 sponges m^−2^). Barrel sponge recruitment was variable and ranged across sites and years from no recruits (Kaledupa Double Spur: 2015, 2016 and Ridge 1, 2015) to 20 recruits (Buoy 1, 2016; Supplementary Fig. 1). Mortality estimates were possible only at Buoy 1 and Sampela 1 and were variable; the highest mortality recorded was 11 individuals in one year (Sampela 1, 2014) and the lowest 3 (Buoy 1, 2015).

**Figure 2:**
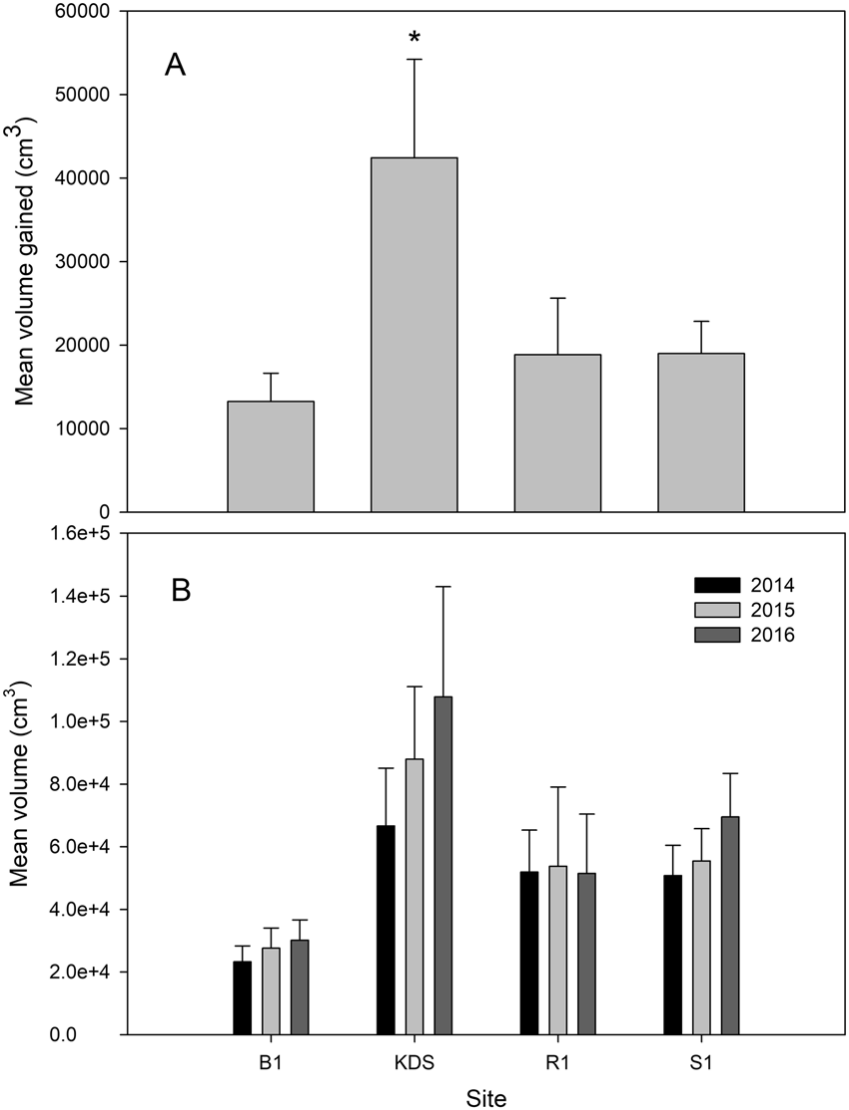
Mean *Xestospongia* spp. volume (cm^3^) at each site (±SE); (**A**) mean volume gained from 2014–2016 for Buoy 1 (B1), Kaledupa Double Spur (KDS), Ridge 1 (R1), and Sampela 1 (S1), and (**B**) mean volume across years at each site. Asterisks (*) and letters denote significant differences between sites.

Mean yearly volume gained varied between sites from 2015–2016 (one-way ANOVA: F_3,220_ = 3.168, *P* = 0.025) and was greatest at Kaledupa Double Spur (41,277 ± 12,479 cm^3^; Fig. 2A). There was no influence of year on mean barrel sponge volume (repeated measures two-way ANOVA, F_2,489_ = 1.124, *P* = 0.326), but it did vary spatially (repeated measures two-way ANOVA, F_1,489_ = 1282.562, *P* < 0.001; Supplementary Table 2; Fig. 2B). Individual sponge volume for B1 (n = 64, 52, 58), KDS (n = 29, 19, 22), R1 (n = 26, 20, 20), and S1 (n = 84, 85, and 67 for 2014, 2015, and 2016, respectively) was highly variable and ranged from 19.99 to 552,937.89 cm^3^ across sites and years. Buoy 1 had the smallest mean sponge volume (23,221 ± 5,082 cm^3^), while the largest was recorded at Karang Gurita (116,721 ± 29,275 cm^3^).

Specific growth was highly variable but was not influenced by location (one-way ANOVA: F_3,117_ = 1.078, *P* = 0.361). Only 5.9% of sponges showed negative SGRs, the remaining were positive. There was a significant negative correlation with initial sponge volume and specific growth rate (r_s_ = −0.476, *P* < 0.01). The mean SGR for across sites was 0.47 ± 0.07 (including negative values), and SGRs were as fast as 6.24 yr^−1^ (Sampela 1) and as slow as −0.12 yr^−1^ (or 0.003 non-negative; Buoy 1 and Sampela 1, respectively). There were instances of tissue loss and partial mortality evident in photographs, often in the form of rubble burial or shearing where up to half of the sponge had been removed (Supplementary Fig. 2). Although there was no direct cause visible in the photographs, the nature of the injuries were suggestive of anchor damage. Tissue loss due to smothering by coral rubble and sedimentation was also common.

### Growth models

The cube root estimates of sponge volume data from 2014 and 2016 were fitted to the difference equations of the growth models, with the model-averaged result revealing a nearly linear relationship for all sites combined (Supplementary Fig. 3). Several different growth models were identified across sites as supported by the Akaike Information Criterion with a correction for sample size (AIC_c_).There was not one clear model of best fit (*w*_*i*_ > 0.9) as the model with the most support, the specialized von Bertalanffy, only had an Akaike weight of 0.533 (Table 1).

**Table 1:** AIC_c_ values across sites ranked by fit following model elimination based on unreliable parameter estimates for specialized von Bertalanffy, generalized von Bertalanffy, Tanaka, and Gompertz models: parameter number in the model (+1) to account for variance (*σ*^2^), residual sum of squares (RSS), bias-corrected Akaike information criterion (AIC_c_), Akaike differences (Δ_*i*_), Akaike weights (*w*_*i*_).

Function	K	RSS	AIC_c_	Δ_*i*_	*w*_*i*_
SvB	3	1803.3	333.1	0	0.533
GvB	4	1790.3	334.4	1.264	0.283
Tanaka	4	1803.3	335.2	2.139	0.183
Gompertz	3	1996.6	345.4	12.323	0.001

### Size-at-age estimates

**Figure 3:**
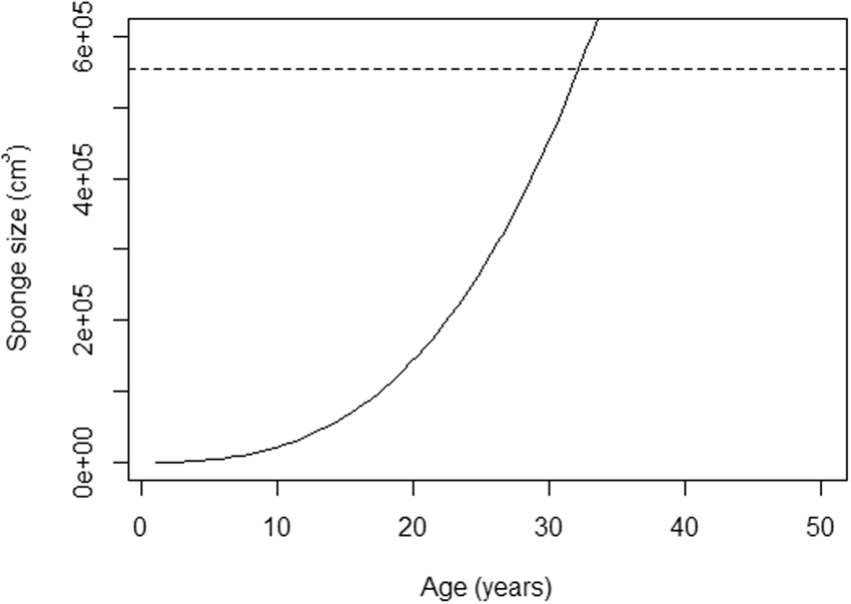
Model averaged size-at-age for all models combined. The dashed line represents the largest sponge volume recorded in this study (552,937.89 cm^3^).

The relationship between volume (cm^3^) at age (years) for all sites combined was determined by predicting size-at-age *t*, and then weighting by *w*_*i*_ to obtain the model averaged estimate of size-at-age *t*. When models of best fit were model averaged and extrapolated to 50 years, the resultant curve was characterized by periods of slow initial growth with a gradual increase over time (Fig. 3). The oldest sponge measured (552,937.89 cm^3^) was estimated to be approximately 33 years old.

### Sponge ages on the USAT Liberty

Sponges ranged in volume from approximately 80,000 cm^3^ to 310,000 cm^3^ (n = 10). Using the model averaged size-at-age curve from the combined Hoga Island sites, the age of the largest sponge measured was estimated to be 27 years old.

## Discussion

Despite the ecological importance of barrel sponges there has only been one previous study quantifying their growth rate in the Caribbean; there are currently no estimates of *Xestospongia* spp. growth rates in the Indo-Pacific. Here we estimated important life history characteristics for Indo-Pacific *Xestospongia* spp. populations. Interestingly, what has been described as the lowest quality site in previous literature (Sampela 1) supported some of the highest sponge densities and mean volumes, yet there was no influence of site on specific growth rate or growth curves. The model averaged estimates of size-at-age using a multi-model inference approach obtained from our study provided considerably lower age estimates for comparable sized barrel sponges in the Caribbean *X. muta*^22^. Therefore, rather than being the Redwoods of the reefs, the faster growth rates of *Xestospongia* spp. in the Indo-Pacific result in a more appropriate comparison to Pines. These results highlight the importance of geographic differences in barrel sponge growth dynamics, and particularly how little is known about the mechanisms driving them.

### Growth of giant barrel sponges

Variability in sponge growth rates is well reported in the literature; some growth is seasonal^21,40,41^, and in some species growth measurements are confounded by shrinkage^40–42^. Previous studies have shown that sponge growth trends vary with environmental quality, though most of this research has emphasized the seasonal effects of temperature^21,22,40,41^. Zero long-term net growth has been reported for large, healthy *Crambe crambe* individuals^24^. Slow seasonal growth rates may be due to resource limitation or necessary regeneration, which may come at the expense of other processes such as growth^43^. It is possible that the timeframe of the present study was not adequate to detect periods of rapid adaptive growth during less stressful conditions.

Surprisingly, *Xestospongia* spp. density and mean volume was high at a site previously characterized by comparatively higher levels of turbidity and decreased light availability (Supplementary Table 2). However, there was no influence of site on specific growth rates or between growth curves, suggesting that despite differences in habitat quality Indo-Pacific *Xestospongia* spp. continue to grow at comparable rates across sites, even in those that might be considered less than optimal (e.g. characterized by low turbidity and sedimentation, etc.). When combined with the lack of influence of depth on mean sponge volume, we propose that barrel sponges at these sites may be less reliant on their photosynthetic symbionts and are mostly feeding heterotrophically. There is evidence that Caribbean *X. muta* share a commensal relationship with their photosynthetic cyanobacteria^44,45^, but this is unknown for Indo-Pacific species. *Xestospongia* spp. in other areas of Sulawesi have been reported to be constrained to deeper depths^46^, supporting a limited reliance on symbionts. Furthermore, recent observations from deeper water sites in the Wakatobi Marine National Park (50–85 m) have found large populations of *Xestospongia* that all appear completely ‘bleached’ but otherwise healthy, suggesting that barrel sponges can survive in almost complete absence of the photosymbionts (Bell unpublished data). An alternative hypothesis is that *Xestospongia* spp. can shift to heterotrophic feeding in conditions less favourable to photosynthesis by its symbionts. The Sampela 1 site is also characterized by elevated chlorophyll-*a* concentrations, previously used as a proxy for potential sponge food sources^47^. It is important to note, however, that the picoplankton ingested by sponges^29^ are likely to have a variety of chlorophyll types, and that dissolved organic matter and heterotrophic picoplankton may provide alternative sources of food. Further research on the feeding biology of *Xestospongia* spp. is necessary to confirm their dependence on photosynthetic symbionts and ability to switch to heterotrophy when light availability is low, as well as how this translates into different growth rates.

The mean specific growth rate (SGR) for the Caribbean barrel sponge *X. muta* was reported as 0.52 ± 0.65 year^−1^ ^22^ (±SD), as compared to 0.47 ± 0.07 year^−1^ (±SE) in the present study. Similarly, Indo-Pacific *Xestospongia* spp. SGR decreased with increasing size. It would therefore be expected that growth over time is influenced by sponge size, and that large sponges would exhibit slower growth. However, while the growth curve in the present study reflects a similar shape to that reported in the Caribbean^22^, Indo-Pacific barrel sponges appear to increase in size more rapidly in the same time frame. Furthermore, there are several potential explanations for the disparity between Caribbean and Indo-Pacific SGRs. While 5.9% of sponges in this study had a negative growth rate, Caribbean sponges exhibited only positive growth. The range of SGRs herein was also larger (−0.12–6.24 yr^−1^ as compared to 0.02–4.04 yr^−1^ in the Caribbean)^22^. The considerable variability in sponge growth within a genus is a common and well recognized trait in many sponge species^20–22,41^. This variability, in conjunction with the temporal difference between sponge surveys (2 years herein as compared to 4.5 in the Caribbean)^22^, may render SGR a less appropriate and robust method of growth estimation in this study. Growth curves, however, allow for the intrinsic variability of inter-individual differences in size^36^. In this instance these analyses, coupled with model selection and MMI based on information theory approach, may reflect higher confidence in growth models over specific growth rate. As both SGRs and growth curves reflect mean growth rates, the conflicting results reported herein highlights the importance of long term monitoring for Indo-Pacific *Xestospongia* spp., as well as the need for further investigation into the parameters which shape the growth of these ecologically important sponges.

The wide range of habitat characteristics present in this study may contribute to differences in mean volume between sites. Abiotic and biotic factors alike are likely to affect the population and individual sponge dynamics at each site; these include factors not measured in this study such as food supply and consumption, hydrodynamics, spatial competition, wide-scale disturbance (e.g. cyclones and disease), and predation, the majority of which is widely understudied in this region (but see^48^). In particular, the importance of spatial competition in shaping the ecology of sponges has received considerable attention^49–54^, and may have a large influence on the dynamics of *Xestospongia* spp. At Sampela 1, for instance, competition with other benthic taxa is reduced than at the other sites as the coral cover is low, which would be expected to influence density, mean volume, and potentially growth rates.

Seasonality in *X. muta* growth was reported in the Caribbean^22^ but was not measured in our study; the volume gained per year in the summer months was in line with growth measured herein from 2014 to 2015 (4,195.53 ± 4,080^21^; 4,572.60 ± 1,394.66 cm^3^, Buoy 1). From 2015–2016, however, the volume gained at Kaledupa Double Spur was over eight-fold larger than the volumes reported in the Caribbean^22^ (40,676.99 ± 12,479.05 cm^3^), though highly variable. The high variation in volume gained among years highlights the importance of interannual variation in environmental conditions as a possible driver of growth variability. Furthermore, the recognized Indo-Pacific *Xestospongia* species complex comprised of cryptic species^26,27^ may also confound measurements of growth. As we were unable to differentiate which species were surveyed, and due to the demographic nature of the study, all sponges were treated at the level of genus. The potential intra-specific variation in sponge growth may have the potential to influence parameters such as volume gained and specific growth rates, although this has not been examined in *Xestospongia* spp. Molecular analyses are required in order to clarify this issue and fully describe the nature of the species complex.

### Model choice and parameter uncertainties

The size-at-age extrapolation used in this study followed multi-model inference (MMI) with a model averaging method commonly employed in other fields to ensure a robust estimation of size at any given age^55^. A distinction of note between this practice and the data presented herein, however, is the ability to “ground truth” using actual size-at-age data, typically otoliths and fish length data^39,55^. As this was not possible in our study, *Xestospongia* size at *t*_0_ was by proxy represented by the smallest sponge in the data^22^. We therefore consider our results to provide conservative growth estimates due to the potential for overestimation of volume at the time of larval settlement. While there were individuals that represented large sponge sites at each site, it is possible that even larger sponges present at these sites were not found and therefore underrepresented, which could affect model fit and size-at-age estimates. Combining the sites for model averaging, however, resulted in a larger sample size (n = 121), which is in line with previous studies^22^. However, based on the results of our size-at-age calculation, and despite the acknowledged sources of potential error we are confident in our age estimations for *Xestospongia*. spp.

Some parameter estimates in the remaining models had moderately large standard errors, likely due to the large parameter number in candidate models (given the apparent linear trend between 2014 and 2016 sizes, Supplementary Fig. 3) or due to the highly variable sponge volumes in the data set. Although the specific values of the parameters estimated by the model fit may therefore be of moderate confidence, their descriptive power can remain unaffected^56^. Employing multi-model averaging is widely considered superior in lieu of *a priori* model choice, but it is possible that our data support another model that we did not examine.

### Redwoods of the reef or Pines of the Indo-Pacific?

Variation in age extrapolated from growth model projections is not uncommon due to differences in model selection, sampling methods, reproductive life history or morphological differentiation^57^; the importance of model choice and parameter uncertainties are detailed in the Supplementary Information. Previous work examining *Xestospongia muta* growth determined the model of best fit by comparing AIC_c_ scores^22^. There was evidence of substantial model support throughout multiple candidate models based on AIC_c_ scores such that estimates were strongly supported^32^. In the same study, a sponge with a volume of 632,912.80 cm^3^ was predicted to be nearly 242 years old. Although the largest sponge measured in the present study was slightly smaller at 552,937.89 cm^3^, and error is expected to increase with the extrapolation of large sponge age, we estimated it to be a maximum of 33 years old based on the model-averaged growth curve (Fig. 3). A more conservative comparison places those in the Indo-Pacific at approximately 22 as compared to between 53 and 55 years of age in the Caribbean for sponges approximately 150,000 cm^3^ in size^22^ (Fig. 3).

Based on our growth models we estimated the largest sponges measured on the USAT Liberty wreck to be approximately 27 years old. As the ship sank in 1963, the maximum age the sponges could be at the time of measurements (2014) is 51 years old. While effort was undertaken to survey a large area, sponges were still chosen haphazardly and it is therefore unlikely that they were the largest and therefore oldest individuals on the wreck. It seems reasonable that sponges would not have recruited immediately to the wreck until suitable biofilms had formed, and therefore the age estimates from the wreck provide strong independent support for our size-at-age estimates in the Wakatobi Marine National Park. In contrast, using the age at size relationships Caribbean *Xestospongia muta* would age the biggest sponge on the wreck at approximately 100 years old, much older than the wreck itself (though caution should be taken in such a direct comparison due to the inherent error associated with *X. muta* age extrapolation)^22^. There are further examples where large sponge size does not correlate with long life in sponges. For example, individuals of *Ianthella basta* nearly 2 m high were reported to be only 10 years old^58^.

### Implications of barrel sponge life-history traits for management and conservation

Previous research on barrel sponges suggests that they should be susceptible to environmental disturbance. They have a large body size, low population connectivity^28^, slow growth, are likely long-lived^22^, and have high larval mortality common to broadcast spawning^59^. However, despite these features we found barrel sponges were larger and more abundant at a low quality site (as determined by published levels of high levels of sedimentation and turbidity and low coral cover, Supplementary Table 2). The much faster growth rates described in this study compared to those reported in the Caribbean may partly explain the high abundance at low quality sites. The reduction in coral cover at Sampela 1 is largely thought to have occurred in the last 10–20 years, with coral cover declining from 30% to <8%^60^, which will likely have released barrel sponges from spatial competition and potentially allowed a larger population size. However, we do not currently have long-term population data on barrel sponges in this region, which would be needed to test this hypothesis.

It is possible that barrel sponges have specific adaptions to live in the sedimented conditions at Sampela 1 that support their success. A recent study found barrel sponges from the Wakatobi Marine National Park to be resilient to the effects of sediment, particularly through the production of mucous, which aids the removal of sediment from the sponge surface^61^. These earlier experimental results combined with our relatively fast growth rate estimates suggest barrel sponges may be more resilient to some environmental impacts, particularly increased sedimentation and turbidity, than might be expected, and may actually benefit from these conditions in some circumstances.

Barrel sponge recruitment might be low and sporadic given the small adult population size and limited connectivity^28^. However, we did find a number of barrel sponge recruits during our study challenging this suggestion, although supply was sporadic. The recruitment rates were generally higher than mortality rates, suggesting populations are not in decline, although recruits would need to be monitored over a longer temporal scale to determine if mortality and recruitment are in equilibrium.

## Conclusions

This study is the first to examine *Xestospongia* spp. demography in the Indo-Pacific as well as to examine the possible role that environmental variation plays in determining size and abundance. Interestingly, results demonstrate that Indo-Pacific barrel sponges achieve a comparable size to that of their Caribbean cohorts much faster, and therefore large barrel sponges on Indo-Pacific reefs are more comparable to Pine trees rather than the Redwoods proposed in the Caribbean^23^. This study highlights how changes in environmental conditions, such as through degradation, may influence these functionally important species. However, barrel sponges also have the potential to dominate in environments where there is low coral abundance. This is particularly the case for sedimented habitats, although there may be energetic costs associated with living in these suboptimal conditions that negatively impact growth rates.

## Electronic supplementary material

Supplementary information

## Data Availability

The datasets generated and analysed during the current study are available from the corresponding author on reasonable request.
