# Author Correction: Growth and longevity in giant barrel sponges: Redwoods of the reef or Pines in the Indo-Pacific?

**DOI:** 10.1038/s41598-019-54609-w

**Published:** 2019-11-27

**Authors:** Emily C. McGrath, Lisa Woods, Jamaluddin Jompa, Abdul Haris, James J. Bell

**Affiliations:** 1 School of Biological Sciences, Victoria University of Wellington, P.O. Box 600, Wellington, 6140 New Zealand; 2 School of Mathematics and Statistics, Victoria University of Wellington, P.O. Box 600, Wellington, 6140 New Zealand; 3 Research and Development Centre on Marine, Coastal and Small Islands, Hasanuddin University, Makassar, Indonesia

Correction to: *Scientific Reports* 10.1038/s41598-018-33294-1, published online 17 October 2018

The original version of this Article contained errors in the Abstract.

“Interestingly, SGRs were slightly slower than that of X. *muta*, yet growth models supported rapid growth; Indo-Pacific sponges were over twice as old as published estimates of comparably sized X. *muta* (53–55 as compared to 23 years of age, respectively), although extrapolation errors are likely to increase with sponge size.”

now reads:

“Interestingly, SGRs were slightly slower than that of X. *muta*, yet growth models supported rapid growth; published estimates of comparably sized X. *muta* were over twice as old as Indo-Pacific sponges (53–55 as compared to 23 years of age, respectively), although extrapolation errors are likely to increase with sponge size.”

These errors have now been corrected in the PDF and HTML versions of the Article.

