# Supplementary material for: Growth and longevity in giant barrel sponges: Redwoods of the reef or Pines in the Indo-Pacific?
Source: Sci Rep. 2018 Oct 17;8:15317. doi: 10.1038/s41598-018-33294-1 (PMC6193018; doi:10.1038/s41598-018-33294-1)
Supplement: Supplementary file 1 — Supplementary information [file 41598_2018_33294_MOESM1_ESM.docx]

# **Growth and longevity in giant barrel sponges: Redwoods of the reef or Pines in the Indo-Pacific?**

EMILY C. M^c^GRATH^1*^, LISA WOODS^2^, JAMALUDDIN JOMPA^3^, ABDUL HARIS^3^, JAMES J. BELL^1^

### *Camera calibration*

### In order to ensure accuracy of three-dimensional photos and subsequent measurements, stereo systems require in-water calibration; this is accomplished using calibration cube photographed in a range of positions^1^. The resultant photographs are analyzed with the CAL software created by J. Seager (<http://www.seagis.com.au>), which calculates camera lens orientation as well as optical and distortion properties^1^.

*Sponge volumetric measurements*

*Xestospongia* spp. volume was calculated by approximating geometric shapes for each sponge. The shapes used and required measurements for each were as follows: cylinder and ovoid cylinder (height, diameter of the top of the sponge as measured from above), barrel (height, top diameter, mid-barrel diameter), sphere (height), inverted truncated elliptical cone (height, top diameter, bottom diameter, area of the top, area of the bottom), and frustrum of a cone (height, bottom diameter). Spongocoel volumes were determined to be either cylinders, ovoid cylinders, or inverted truncated elliptical cones as appropriate. Parameter measurements were incorporated into the appropriate formulae and corrected for spongocoel volume^2^.

*Summary of environmental variables*

Supplementary Table 1. Environmental parameters for Hoga Island sites. NTU refers to nephelometric turbidity units, STU refers to standard turbidity units.

| Source | Year collected | Variable | Units | Site | | | | Error (±) |
| --- | --- | --- | --- | --- | --- | --- | --- | --- |
|  |  |  |  | Sampela 1 | Buoy 1 | Kaledupa Double Spur | Ridge 1 |  |
| ***abiotic*** | | | | | | | | |
| Rowley (2014) | 2009 | Temperature | ºC | 25.61-29.36 | -- | -- | 24.06-28.07 | SE |
| Powell (2014) | 2010 |  |  | 27.73 ± 0.12 | 27.37 ± 0.13 | 27.92 ± 0.21 | 27.66 ± 0.13 | SD |
| Rowley (2014) | 2009 | Salinity | PSU | 32.5 ± 0.45 | -- | -- | 32.6 ± 0.26 | SE |
| Crabbe and Smith (2002) | 2001 |  | ppt | 32 | -- | 34 | -- | SD |
| Rowley (2014) | 2009 | Flow | m s^-1^ | 0.05 ± 0.022 | -- | -- | 0.031 ± 0.026 | SE |
| Powell (2014) | 2010 |  |  | 0.063 ± 0.044 | 0.022 ± 0.008 | 0.038 ± 0.041 | 0.04 ± 0.046 | SD |
| Powell (2014) | 2010 | Substrate angle | º | 46.67 ± 31.09 | 58.33 ± 25.43 | 62.83 ± 33.86 | 65.0 ± 21.68 | SD |
| Crabbe and Smith (2002) | 2001 | Rugosity index |  | 13.94 ± 0.95 | -- | 20.06 ± 1.85 | -- | SD |
| Rowley (2014) | 2009 | Light | K_d(PAR)min-max_ | 0.31-3.14 | -- | -- | 0.1-1.56 | SE |
| Hennige (2008) | 2005 |  |  | 0.31 | -- | -- | -- | SE |
| Hennige (2010) | 2007-2008 |  |  | 0.20 ± 0.02 | -- | -- | -- |  |
| Biggerstaff (2016) | 2013-2015 | PAR | µmol photons m^-2^ s^-1^ | 229.2 ± 7.5 |  |  |  | SE |
| Powell (2014) + herein | 2010, 2014-2016 | Chlorophyll-*a* | µg l^-1^ | 2.42 ± 0.49 | 0.31 ± 0.08 | 2.68 ± 0.99 | 1.30 ± 0.32 | SE |
| Rowley (2014) | 2009 |  |  | 0.3 ± 0.01 | -- | -- | 0.35 ± 0.03 | SE |
| Powell (2014) | 2010 |  |  | 0.39 ± 0.1 | 0.25 ± 0.13 | 0.26 ± 0.14 | 0.33 ± 0.08 | SD |
| Powell (2014) + herein | 2010, 2014-2016 | Turbidity | NTU | 4.62 ± 0.77 | 2.98 ± 0.89 | 2.07 ± 0.39 | 2.11 ± 0.22 | SE |
| Biggerstaff (2016) | 2013-2015 |  |  | 6.596 ± 1.099 (5m) |  |  |  | SE |
|  |  |  |  | 3.232 ± 1.055 (9m) |  |  |  | SE |
| Rowley (2014) | 2009 |  | STU | 4.38 ± 1.80 | -- | -- | 0.17 ± 0.33 | SE |
| Powell (2014) | 2010 |  |  | 3.88 ± 4.58 | 1.45 ± 0.69 | 0.78 ± 0.38 | 0.19 ± 033 | SD |
| Powell (2014) + herein | 2010, 2014-2016 | Sediment | g dry weight day^-1^ | 0.26 ± 0.04 | 0.21 ± 0.056 | 0.109 ± 0.05 | 0.112 ± 0.022 | SE |
| Crabbe and Smith (2002) | 2001 |  |  | 0.2 ± .06 | -- | -- | 0.05 ± 0.002 | SD |
| Rowley (2014) | 2009 |  |  | 0.33 ± 0.03 | -- | -- | 0.12 ± 0.007 | SE |
| Powell (2014) | 2010 |  |  | 0.357 ± 0.168 | 0.162 ± 0.029 | 0.109 ±0.047 | 0.112 ± 0.022 | SD |
| Salinas de Leon (2011) | 2008, 2009 |  |  | 0.23 ± 0.04 | -- | -- | -- | SE |
| Biggerstaff (2016) | 2013-2015 |  |  | 0.221 ± 0.03 | -- | -- | -- | SE |
| ***biotic*** | | | | | | | | |
| McMellor (2007) | 2002 | Hard coral | % cover | 32.6 ± 2 |  | 59.5 ± 2 | 53.5 ± 1.2 | SE |
|  | 2003 |  |  | 28.5 ± 1.8 |  | 50.5 ± 0.5 | 53.3 ± 0.3 | SE |
|  | 2004 |  |  | 24.6 ± 1.3 |  | 51.5 ± 3.5 | 52.3 ± 0.5 | SE |
|  | 2005 |  |  | 12.2 ± 1.6 |  | 28 ± 1.5 | 29 ± 4 | SE |
|  | 2006 |  |  | 12.1 ± 1.7 |  | 29 ± 1.5 | 22.3 ± 3 | SE |
|  | 2007 |  |  | 12.2 ± 1.6 |  | 25.5 ± 1.4 | 23 ± 3.1 | SE |
|  | 2002 | Coral rubble |  | 11 ± 1.2 |  | 5 ± 1.1 | 3 ± 0.8 | SE |
|  | 2003 |  |  | 10.2 |  | 6.5 | 3 | SE |
|  | 2004 |  |  | 10 ± 1.0 |  | 6.8 ± 2.5 | 3 ± 0.8 | SE |
|  | 2005 |  |  | 27.5 ± 4 |  | 3.5 ± 2.5 | 4.9 ± 0.9 | SE |
|  | 2006 |  |  | 27.4 ± 3.9 |  | 22 ± 3.4 | 15 ± 1.3 | SE |
|  | 2007 |  |  | 24.3 ± 0.6 |  | 21.5 ± 1.7 | 10 ± 5 | SE |
|  | 2002 | Macroalgae |  | 35 ± 5 |  | 20 ± 2.1 | 23.5 ± 4 | SE |
|  | 2003 |  |  | 31.5 ± 0.15 |  | 19.8 ± 0.05 | 27.4 ± 0.5 | SE |
|  | 2004 |  |  | 32.5 ± 0.3 |  | 19.7 ± 1.8 | 14 ± 0.8 | SE |
|  | 2005 |  |  | 4.8 ± 1.2 |  | 7 ± 1.2 | 7.5 ± 1.9 | SE |
|  | 2006 |  |  | 1.7 ± 0.2 |  | 6.8 ± 1.5 | 4.8 ± 1.1 | SE |
|  | 2007 |  |  | 14.2 ± 0.25 |  | 6.1 ± 1.4 | 4.9 ± 1.5 | SE |
| Powell et al. (2014) | 2010 | Hard coral |  | 11.11 ± 7 | 23.36 ± 11.26 | 27.49 ± 14.86 | 35.7 ± 13.62 | SD |
|  |  | Soft coral |  | 6.39 ± 5.44 | 4.96 ± 3.03 | 13.08 ± 8.39 | 17.34 ± 16.96 | SD |
|  |  | Coralline algae |  | 14.38 ± 15.53 | 20.81 ± 11.21 | 27.43 ± 11.75 | 12.23 ± 10.66 | SD |
|  |  | Other (non-coralline algae) |  | 6.8 ± 6.8 | 4.18 ± 2.44 | 2.97 ± 2.44 | 6.86 ± 5.02 | SD |
| Rowley (2014) | 2009 | Hard coral |  | 2.33 ± 2.04 |  |  | 40.12 ± 3.1 | SE |
|  |  | Dead coral/rubble |  | 38.34 ± 7.1 |  |  | 6.96 ± 1.27 | SE |
|  |  | Soft coral |  | 3.88 ± 1.42 |  |  | 38.98 ± 3.83 | SE |
|  |  | Biotic |  | 4.31 ± 1.21 |  |  | 6.99 ± 1.44 | SE |
|  |  | Abiotic |  | 48.14 ± 6.3 |  |  | 6.95 ± 1.9 | SE |
| Salinas de Leon (2013) | 2008-2009 | Hard coral |  | 14 ± 1.71 |  |  |  | SE |
|  |  | Bare substratum |  | 24 ± 3.4 |  |  |  | SE |
|  |  | Coral rubble |  | 28.2 ± 5.59 |  |  |  | SE |
| Bell and Smith (2004) | 2002 | Sponges |  | 29.5 ± 7 |  |  |  | SE |
| Haapkylä et al. (2009) | 2005 | Hard coral |  | 12.33 ± 1.5 |  |  |  | SE |
|  | 2007 |  |  | 12.2 ± 2 |  |  |  | SE |
| Henninge et al. (2008) | 2005 | Hard coral |  | 31.7 ± 7.90 |  |  |  | SE |
| Crabbe et al. (2005) | 2001-2002 | Hard coral (10m) |  | 6.99 ± 1.41 |  |  |  | SD |
|  |  | Hard coral (5m) |  | 7.33 ± 0.94 |  |  |  | SD |
|  |  | Hard coral (reef flat) |  | 4.66 ± 3.09 |  |  |  | SD |
| Haapkylä et al. (2007) | 2005 | Hard coral (crest) | Mean coral cover | 0.31 |  |  |  | SD |
|  |  | Hard coral (flat) |  | 0.3 ± 0.8 |  |  |  | SD |
|  |  | Hard coral (slope) |  | 0.38 ± 0.4 |  |  |  | SD |

*Candidate growth models*

The biological meaning for each parameter associated with each function is as follows^3^: S_∞_ is the size (volume) reached after an infinite growth period, K equates to the growth rate, t_0_ is the theoretical size at time 0, t* is the age of growth inflection, and D determines the shape of the curve (in most cases approximately sigmoid). The Tanaka growth model for indeterminate growth includes the following parameters^3^: *a* is related to maximum growth rate (~1/*a*^0.5^), *c* is the age at which growth is maximum, *d* shifts body size at which growth is maximum, and *f* is the measure of rate of change of the growth rate (Supplementary Table 1).

Supplementary Table 2. Difference equations for candidate growth models.

| Function name | Function | Parameters |
| --- | --- | --- |
| Specialized von Bertalanffy | $S_{2}=S_{1}+\left( S_{\infty}-S_{1} \right)*(1-e^{-K*dt})$ | S_∞_, K, t_0_ |
| Generalized von Bertalanffy | $S_{2}={{(S}_{\infty}}^{1/D}*\left( 1-e^{-K*dt} \right)+{S_{1}}^{1/D}*\left( e^{-K*dt} \right))^{D}$ | S_∞_, K, t_0_, D |
| Gompertz | ${ln(S}_{2})=\ln\left( S_{\infty} \right)*\left( 1-e^{-K*dt} \right)+\ln\left( S_{1} \right)*(e^{-K*dt})$ | S_∞_, K, t^*^ |
| Richards | $S_{2}={{(S}_{\infty}}^{-1/D}*\left( 1{-e}^{-K*dt} \right)+{S_{1}}^{-1/D}*(e^{-K*dt}))^{-D}$ | S_∞_, K, t^*^,D |
| Tanaka | $S_{2}=1/(f^{0.5})*ln(2*G+2*\left( G^{2}+f*a)^{0.5} \right)+d$ $G=E/4-f*a/E+f$  $E=exp[\left( f^{0.5}*\left( S_{1}-d \right) \right)]$ | a, c, d, f |

*Akaike Information Criterion and multi-model inference*

Model fit and biological accuracy of parameter estimates were examined with an information theory approach. This method entails consideration of all candidate models, their parsimony with Akaike’s Information Criterion (AIC), and the accuracy of parameter estimation^4^. This approach is reliant on the strength of evidence in the data and thereby more relevant than classically used tools such as R^2^ ^5,6^. The bias-corrected Akaike Information Criterion (AIC_c_) was utilized to compare growth models as the ratio of sample size to parameter number (n/k) was less than 40^7,8^ (Supplementary Eq. 1).

*Supplementary Eq. 1*

$${AIC}_{c}=AIC+ {2k(k+1)}/{(n-k)-1}$$

Akaike weights (*w_i_*) represent the relative likelihood to the candidate model of best fit^9^. In the event that data have a *w_i_* above 0.9, the model in question is deemed the only appropriate model for the dataset. Should the Akaike weight (*w_i_*) support more than one model, uncertainty in model selection cannot be ignored^6,9^ and multi-model inference (MMI) using model averaging should be considered^4^. Model averaging weighs potential uncertainty in each candidate model in order to make more confident inferences and estimates the predicted response variable from multiple candidate models^4^.

## *Barrel sponge demography*

Supplementary Fig. 1. Recruit counts per site over time: Buoy 1 (B1), Sampela 1 (S1), Kaledupa Double Spur (KDS), Ridge 1 (R1).

Supplementary Table 3. Spatial variation in mean volume (cm^3^) across each site (repeated measures two-way ANOVA; Buoy 1, Sampela 1, Kaledupa Double Spur, Ridge 1) and over time (2014, 2015, 2016). Asterisks (*) denote a significant effect (*P* < 0.05).

| Source | df | MS | F | *P* |
| --- | --- | --- | --- | --- |
| **Tests of Within-Subjects Effects** | | | | |
| Corrected Model | 5 | 2064.407 | 8.602 | < 0.001* |
| Volume x Site | 1 | 397820.2 | 1282.562 | < 0.001* |
| Volume x Year | 2 | 269.872 | 1.124 | 0.326 |
| **Tests of Between-Subjects Effects** | | | | |
| Site | 3 | 3339.418 | 13.914 | < 0.001* |
| Error | 489 | 240.004 |  |  |
|  | Tukey *post-hoc* tests | | Difference | *P* |
|  |  |  |  |  |
|  | B1 - KDS | | -13.6 | < 0.001* |
|  | B1 - R1 | | -7.03 | 0.005 |
|  | B1 - S1 | | -7.75 | < 0.001* |
|  | KDS - R1 | | 6.57 | 0.028 |
|  | KDS - S1 | | 5.85 | 0.011 |
|  | R1 - S1 | | -0.71 | 0.764 |

Supplementary Fig. 2. Sponge mortality over time; damage may be attributed to burial by sediment and rubble (1a-c), tissue necrosis (2a-c), and possible anchor shearing (3a,b). Scale bars = 10.4 cm.


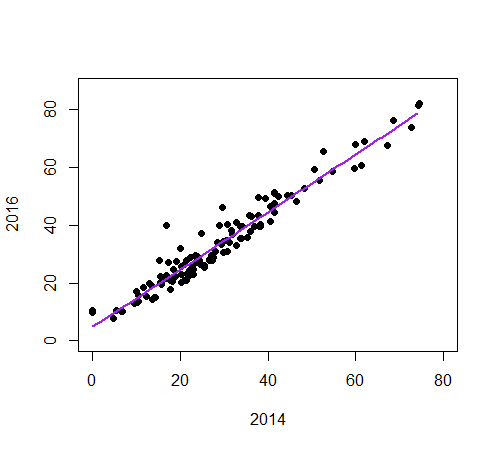


Supplementary Fig. 3. Walford plot of initial sponge volume (2014) and final volume (2016). Symbols represent cube root transformed volume data and the corresponding line represents the size in 2016 predicted by 2014 data using the model-average result of the supported difference equations.

Supplementary Table 4. Parameter estimates for candidate models.

|  | Gompertz | | Specialized | | Generalized von | | | Tanaka | | |
| --- | --- | --- | --- | --- | --- | --- | --- | --- | --- | --- |
|  |  |  | von Bertalanffy | | Bertalanffy | | |  |  |  |
|  | S_∞_ | K | S_∞_ | K | S_∞_ | K | D | *a* | *d* | *f* |
|  | 2065.8 | 1.68E-02 | 3421.5 | 7.31E-04 | 5942.4 | 2.72E-04 | 0.912 | 0.117 | 6620.1 | 1.37E-06 |

**References**

1. Abdo, D. A. *et al.* Efficiently measuring complex sessile epibenthic organisms using a novel photogrammetric technique. *J. Exp. Mar. Bio. Ecol.* **339,** 120–133 (2006).

2. McMurray, S. E., Blum, J. E. & Pawlik, J. R. Redwood of the reef: growth and age of the giant barrel sponge Xestospongia muta in the Florida Keys. *Mar. Biol.* **155,** 159–171 (2008).

3. Brey, T. Population dynamics in benthic invertebrates. A virtual handbook. *http://www. awi-bremerhaven. de/Benthic/Ecosystem/FoodWeb/Handbook/main. html. Alfred Wegener Institute for Polar and Marine Research, Germany* (2001).

4. Katsanevakis, S. & Maravelias, C. D. Modelling fish growth: multi‐model inference as a better alternative to a priori using von Bertalanffy equation. *Fish Fish.* **9,** 178–187 (2008).

5. Diouf, K. *et al.* Effects of the environment on fish juvenile growth in West African stressful estuaries. *Estuar. Coast. Shelf Sci.* **83,** 115–125 (2009).

6. Katsanevakis, S. Modelling fish growth: model selection, multi-model inference and model selection uncertainty. *Fish. Res.* **81,** 229–235 (2006).

7. Shono, H. Efficiency of the finite correction of Akaike’s Information Criteria. *Fish. Sci.* **66,** 608–610 (2000).

8. Symonds, M. R. E. & Moussalli, A. A brief guide to model selection, multimodel inference and model averaging in behavioural ecology using Akaike’s information criterion. *Behav. Ecol. Sociobiol.* **65,** 13–21 (2011).

9. Burnham, K. P. & Anderson, D. R. *Model selection and multimodel inference: a practical information-theoretic approach*. (Springer Science & Business Media, 2003).
